# Piezoelectric Transducers for Structural Health Monitoring of Joint Structures in Cylinders: A Wave-Based Design Approach

**DOI:** 10.3390/s20030601

**Published:** 2020-01-21

**Authors:** Wenjun Wang, Lin Li, Yu Fan, Zhou Jiang

**Affiliations:** 1 School of Energy and Power Engineering, Beihang University, Beijing 100191, China; zy1704109@buaa.edu.cn (W.W.); feililin@buaa.edu.cn (L.L.); sy1404110@buaa.edu.cn (Z.J.); 2 Beijing Key Laboratory of Aero-Engine Structure and Strength, Beijing 100191, China

**Keywords:** piezoelectric transducer, structural health monitoring, joint structures, wave-mode conversion, wave and finite element method

## Abstract

Joint structures, such as riveting, hinges, and flanges, are widely used in complex mechanical systems. A small unexpected change of a joint can lead to complicated wave-scattering in its connected waveguides. The conversion between wave modes can be used to quantify the variation of the connection status of joints. This gives rise to the challenge of exciting and sensing only one specific wave mode in practice. In this paper, transmitted wave amplitudes of a flange joint are first calculated by the wave finite element method (WFEM) to study the quantitative relationship between the local stiffness changes of the damaged site and the wave-mode conversion. Wave-mode piezoelectric transducers are subsequently designed for torsional, longitudinal, and flexural waves in cylindrical waveguides. The idea is to use the distribution and interconnection of the piezoelectric materials to cancel the charge contributed from the non-targeting waves. We conducted numerical simulations to demonstrate the selective coupling features of the designed wave transducers and found difference of several orders of magnitude in voltages between targeting wave mode and other wave modes. Four selected wave transducers were then extended to monitor the connection status of the flange. The wave-scattering features in the simulation and WFEM were verified to be in good agreement.

## 1. Introduction

Large-scale mechanical systems such as spacecraft contain thousands of simple components connected by joint structures. Due to complex operating conditions, these joint structures may undergo contact surface oxidation, thermal deformation, preload reduction, and other minor local changes, which can affect the dynamic properties of the entire mechanical system [1]. Structural health monitoring (SHM) is therefore crucial to ensure structural safety.

Three types of SHM techniques were successively developed in the last two decades for general structural systems: (1) vibration-based SHM; (2) SHM based on electromechanical impedance; and (3) guided-wave-based SHM. Vibration-based SHM techniques [2,3] detect changes of structures in natural frequencies, modal shapes, or dynamic responses to identify local damage. By attaching piezoelectric transducers to the monitored portion, the impedance of the piezo-materials is verified to be reasonably sensitive to the dynamic status of the monitored site [4,5]. Although these two methods were extended to identify the connected status of a single bolt [6], they have some inherent limitations when applied to joint structures. First, the local damage of joint structures commonly causes minor changes in the dynamic characteristics of entire structures, such as modal frequencies, thus the diagnostic changes of the damages can be masked by other changes of the structural system. Second, to verify that the changes of the overall dynamic characteristics are indeed caused by the joint structure requires additional measurements, such as the modal shapes or their curvature [7]. This increases the complexity of sensing the local damage to the joint structure, hence the sensitivity of the above methods may be unsatisfied.

Most promising for joint structures is probably guided-wave-based SHM, which has attracted considerable attention. It has several inherent advantages: (1) a small wavelength ensures sufficient interaction of the guided waves and local minor damage; (2) the excitation frequency of guided waves can be very high, hence signals of the operating and ambient frequencies barely perturb transmitted signals of guided waves; (3) the characteristics of piezoelectric materials confer on guided waves the capability of a wide sweep range. For these reasons, we develop wave-based approaches for SHM of joint structures in this work.

In wave-based SHM approaches, transducers are generally arranged at two ends of the structure. One is to actuate guided waves, and the other to receive the excited guided waves. Features of guided waves, such as velocity, attenuation, scattering, and mode conversion, are used to indicate changes in structural dynamics. Du et al. [8,9] employed a modified time reversal method for a single bolt joint and demonstrated that the proposed tightness index can indicate the joint status of the bolt. Rhee et al. [10] preliminarily explored the application of Lamb waves to characterize the joint status of a bolt array in a plate, and concluded the experience of bolt modeling and piezoelectric transducer bias. The information carried by a single wave can barely characterize multiple bolts due to the complexity of the connection state. The wave-mode conversion between various waves subsequently drew researchers’ attention.

The wave-mode conversion indicates that part of the energy from a propagating wave converts to a previously nonexistent wave-mode. Lee et al. [11] simulated the interaction between Lamb waves and damage in a metallic plate. They found that the damage can cause transmission, reflection, and mode conversion (from A0 to S0) of Lamb waves. Fromme et al. [12] analyzed the wave-mode conversion when a Lamb wave transmitted through a circular damage hole. The results showed that the amplitude of the S0 mode converted from A0 mode has a strong correlation with the depth of the hole. Willberg et al. [13] investigated the laminated composited plates that were widely used in the aerospace industry and demonstrated that continuous mode transitions occur due to the asymmetry of layered materials. Li et al. [14] demonstrated that a slight asymmetry can lead to wave-mode conversion in a cylindrical shell. This literature shows that wave-mode conversion characteristics can be a promising indicator to monitor slight local damage.

Researchers have also developed analytical and numerical methods to study the interaction between damage and wave-mode conversion. Zhang et al. [15] decomposed the frequency-domain response of a cable into an analytical four-wave basis and used the evanescent wave amplitudes induced by discontinuities as indicators of damage. Ichchou et al. [16] developed WFEM with the diffusion matrix method (DMM) and analyzed the wave-mode conversion of a block coupling structure with a notch. They concluded that high-order modes were more sensitive to small notch junctions. Huang et al. [17] investigated the wave-scattering features of a thin-wall structure with attached piezoelectric patches by DMM. The results demonstrated that the shunted piezoelectric patches can be used to control wave propagation in a thin-wall structure. Gallezot et al. [18] presented a perfectly matched layer (PML)-based hybrid method for the numerical modeling of wave-scattering by inhomogeneities in open waveguides. They concluded the experience for choosing model representation from three modes: trapped, leaky, and PML modes.

Numerical tools for wave-scattering have been fully developed. However, in practice, it is challenging to measure the conversion among waves. Droz et al. [19] generated near-cut-on guided resonances (GRs) in a composite plate using an array of piezoelectric transducers. Numerical and experimental results showed that a GR 8 wave pulse was excited by the transducers’ array. Furusawa et al. [20] designed a circumferentially distributed electromagnetic transducer for longitudinal and torsional waves in cylinders. Kharrat et al. [21] presented circumferentially distributed piezoelectric actuators for the first-order torsional wave in cylindrical shells. While transducers designed according to the above literature can actuate certain low-order cylindrical waves, a general design approach for all types and orders of waves in cylinders is lacking. Moreover, verification has not been conducted to guarantee the excited guided wave is single-mode, which limits the accuracy of detecting wave-mode conversion.

In this work, we use the proposed wave-mode transducers to detect the wave-mode conversion induced by the local stiffness changes of a damaged site. First, the wave-mode conversion features induced by these changes are studied by WFEM. To detect this conversion, a design approach of piezoelectric wave-mode transducers in cylinders is proposed. Second, numerical studies of the finite element method (FEM) are subsequently conducted to verify such features of transducers. Finally, four selected transducers are applied to monitor the wave-scattering characteristics of a drum-flange structure. The simulated results verify the features of transmitted waves demonstrated in WFEM.

## 2. Problem Formulation

**Figure 1:**
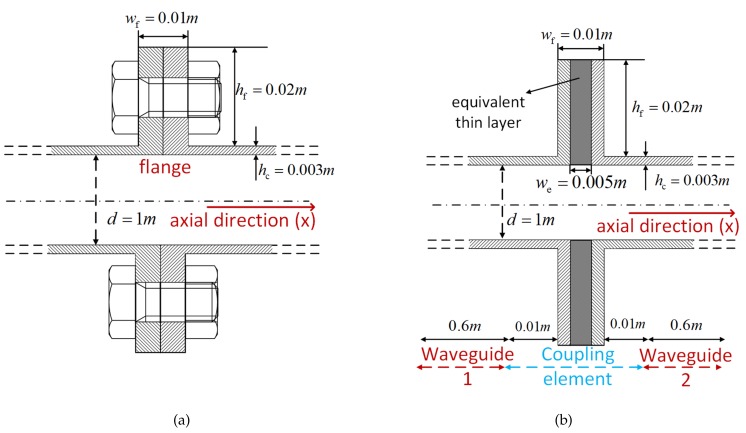
Drum-flange structure of an aero-engine. (**a**) section drawing; (**b**) simplified model with equivalent thin layer.

Figure 1a illustrates a diagrammatic cross-section of a drum-flange structure mounted on an aero-engine casing. As shown in Figure 1b, the equivalent thin-layer element method [22] is employed to model the flange joint. This method accelerates the modeling process and retains the dynamic characteristics and cyclic symmetry of the flange, which satisfies the research needs in this work. The geometric features are assumed to be d=1m, hc=0.003mhchcdd≪11/4040, $h_{f}=0.02\;m$, $w_{f}=0.01\;m$, and $w_{e}=0.005\;m$. The coupling element consists of the thin layer and cylindrical shell of 0.01-m thickness placed on each side, which ensures the continuity of the waveguide and the coupling element. Waveguides 1 and 2 are cylindrical shells of 0.6 m.

Suppose small damage appears in the flange, such as wear, corrosion, oxidation, or a loosened bolt, and these damages always lead to stiffness loss of the damaged position. The reduction of the elastic modulus of the equivalent thin-layer elements in $0^{\circ }$ to $15^{\circ }$ is to simulate this local dynamic change induced by the small damage. Two extents, 10% and 50%, of elastic modulus reduction are considered. The former is to verify the effectiveness of the wave-mode conversion to identify slight damage, and the latter is to study the wave-scattering features of the local damage. These simulated damages inevitably lead to different wave-mode conversions in waveguide 2, in contrast to an undamaged flange. Hence, we first investigate the dispersive curves and mode shapes of the guided waves in a cylindrical waveguide, and for versatility, employ the WFEM numerical method, which can be extended to calculate other cross sections of waveguides (e.g., rectangular). Then, we induce DMM to distinguish the conversion among these waves caused by the local damage.

To detect wave-mode conversion in practice, our core innovation is the piezoelectric wave-mode transducer, whose design is applicable to both low- and high-order torsional, longitudinal, and flexural waves. The transducer is only coupled with the targeting wave, and has very low interaction with other waves. This strong selective coupling behavior is achieved by setting different polarization directions, connection patterns, and distributions of piezoelectric materials according to the orthogonality of waves.

Numerical simulation in FEM aims to verify the selective features of transducers. By implementing transducers on both ends of a cylinder as actuators/sensors and applying exciting voltages on actuators, the voltage responses of the sensors indicate coupling/decoupling levels with targeted/undesired wave modes.

Four selected transducers are extended to monitor the connection status of a flange in Figure 1b. The simulation results verify the wave-scattering features obtained in WFEM.

## 3. Numerical Tools to Analyze Wave-Scattering in 1D Waveguides

### 3.1. Wave and Finite Element Method

WFEM relies on the periodicity of the waveguide geometry, and only needs to model a periodic cell of the waveguide in commercial FEM software [23]. It yields the relationship between the wave number *k* and the circular frequency ω, i.e., the dispersive curves, as well as the wave shapes [24]. For a partly periodic structure, such as with both periodic and non-periodic parts, this method can also be applied through inducing DMM [16]. Time consumption and matrix ill-conditioning increase rapidly due to many cross-sectional degree of freedoms (DOFs). Efforts to overcome this include: (1) fixed- and free-interface component modal synthesis (CMS) methods [25]; (2) an enhanced eigenvalue scheme [26]; and (3) multi-scale adaption strategies [27]. Moreover, this method can be employed for both 1D and 2D waveguides [28,29]. In this work, we employ 1D-WFEM so that this procedure is applicable to an arbitrary cross-section (e.g., ring/rectangular). For clarity, we introduce the details of WFEM for 1D waveguides.

**Figure 2:**
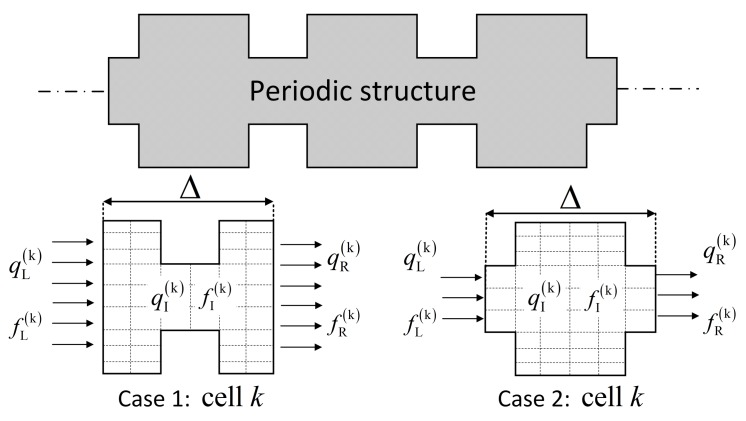
One-dimensional periodic structure. Cases 1 and 2 represent two arbitrary periodic substructures.

As shown in Figure 2, a one-dimensional periodic structure can be arbitrarily discretized into case 1 or case 2. The dynamic stiffness matrix D of cell *k* is

(1) $$\left(-\omega^{2}M+j\omega C+K\right)q=\left[\begin{array}{ccc} D_{\mathrm{LL}} & D_{\mathrm{LR}} & D_{\mathrm{LI}}\\ D_{\mathrm{RL}} & D_{\mathrm{RR}} & D_{\mathrm{RI}}\\ D_{\mathrm{IL}} & D_{\mathrm{IR}} & D_{\mathrm{II}} \end{array}\right]q=f,$$

where q is the displacement vector and f is the force vector, and the subscripts L, R, and I represent left DOFs, right DOFs, and internal DOFs, respectively. For brevity, the harmonic time dependence $e^{j\omega t}$ is suppressed in this work. Assuming no load is applied on internal DOFs, Equation (1) is condensed to

(2) $$\left[\begin{array}{cc} {\tilde{D}}_{\mathrm{LL}} & {\tilde{D}}_{\mathrm{LR}}\\ {\tilde{D}}_{\mathrm{RL}} & {\tilde{D}}_{\mathrm{RR}} \end{array}\right]\left[\begin{array}{c} q_{L}\\ q_{R} \end{array}\right]=\left[\begin{array}{c} f_{L}\\ f_{R} \end{array}\right],$$

where

$$\left[\begin{array}{cc} {\tilde{D}}_{LL} & {\tilde{D}}_{LR}\\ {\tilde{D}}_{RL} & {\tilde{D}}_{RR} \end{array}\right]=\left[\begin{array}{cc} D_{LL} & D_{LR}\\ D_{RL} & D_{RR} \end{array}\right]-\left[\begin{array}{c} D_{\mathrm{LI}}\\ D_{\mathrm{RI}} \end{array}\right]D_{\mathrm{II}}^{-1}\left[\begin{array}{cc} D_{\mathrm{IL}} & D_{\mathrm{IR}} \end{array}\right]$$

The standard Craig-Bampton modal synthesis method is introduced to accelerate the calculation of $D_{\mathrm{II}}^{-1}$. By introducing the Bloch theorem [30], one can obtain that

(3) $$\left(T-\lambda I\right)\left[\begin{array}{c} q_{L}\\ f_{L} \end{array}\right]=0,$$

where

$$T=\left[\begin{array}{cc} -{\tilde{D}}_{\mathrm{LR}}^{-1}{\tilde{D}}_{\mathrm{LL}} & {\tilde{D}}_{\mathrm{LR}}^{-1}\\ -{\tilde{D}}_{\mathrm{RL}}+{\tilde{D}}_{\mathrm{RR}}{\tilde{D}}_{\mathrm{LR}}^{-1}{\tilde{D}}_{\mathrm{LL}} & -{\tilde{D}}_{\mathrm{RR}}{{\tilde{D}}^{-1}}_{\mathrm{LR}} \end{array}\right]$$

is the transfer matrix of a unit cell [31], and $\lambda =e^{-jk\Delta }$ indicates the phase and amplitude changes when guided waves transmit through this cell. Δ is the length of the one-dimensional periodic substructure and *k* is the wavenumber. Directly conducting eigenvalue decomposition on T can induce a numerical problem. The format [26]

(4) $$\left(\left[\begin{array}{cc} 0 & \sigma I\\ -{\tilde{D}}_{\mathrm{RL}} & -{\tilde{D}}_{\mathrm{RR}} \end{array}\right]-\lambda \left[\begin{array}{cc} \sigma I & 0\\ {\tilde{D}}_{\mathrm{LL}} & {\tilde{D}}_{\mathrm{LR}} \end{array}\right]\right)\left(\begin{array}{c} q_{L}\\ q_{R} \end{array}\right)=0$$

has the same eigenvalue as Equation (3) and performs a better condition number, where

$$\sigma =\frac{{∥{\tilde{D}}_{\mathrm{RR}}∥}_{2}}{N^{2}}$$

is introduced to balance the magnitudes of the dynamic matrix and the identity matrix I, and *N* is the number of left DOFs. The eigenvalues occur in pairs as $\lambda_{i}^{+}$ and λi-=11λi+λi+, corresponding respectively to positive and negative waves. However, the eigenvector obtained in Equation (4) is ${\left[\begin{array}{cc} \phi_{q} & \lambda \phi_{q} \end{array}\right]}^{T}$. It must be post-processed by Equation (2) into ${\left[\begin{array}{cc} \varphi_{q} & \varphi_{f} \end{array}\right]}^{T}$, where

(5) $$\varphi_{f}={\tilde{D}}_{\mathrm{LL}}\varphi_{q}+\lambda {\tilde{D}}_{\mathrm{LR}}\varphi_{q}.$$

This is also the eigenvector of the transfer matrix T. Conducting the same procedure, one can obtain the left and right eigenvector matrices,

(6) $$\begin{array}{cc} \Theta =\left[\begin{array}{cc} \Theta_{q}^{+} & \Theta_{f}^{+}\\ \Theta_{q}^{-} & \Theta_{f}^{-} \end{array}\right] & \Phi =\left[\begin{array}{cc} \Phi_{q}^{+} & \Phi_{q}^{+}\\ \Phi_{f}^{-} & \Phi_{f}^{-} \end{array}\right] \end{array},$$

where ${\Phi_{q}}^{+}$ and ${\Phi_{q}}^{-}$ are displacement vectors of positive and negative waves, respectively, and ${\Phi_{f}}^{+}$ and ${\Phi_{f}}^{-}$ are the respective force vectors of positive and negative waves. These are the same for Θ. Normalized formats for Θ and Φ can be obtained by

(7) ΘΦ=I.

### 3.2. Diffusion Matrix Method

**Figure 3:**
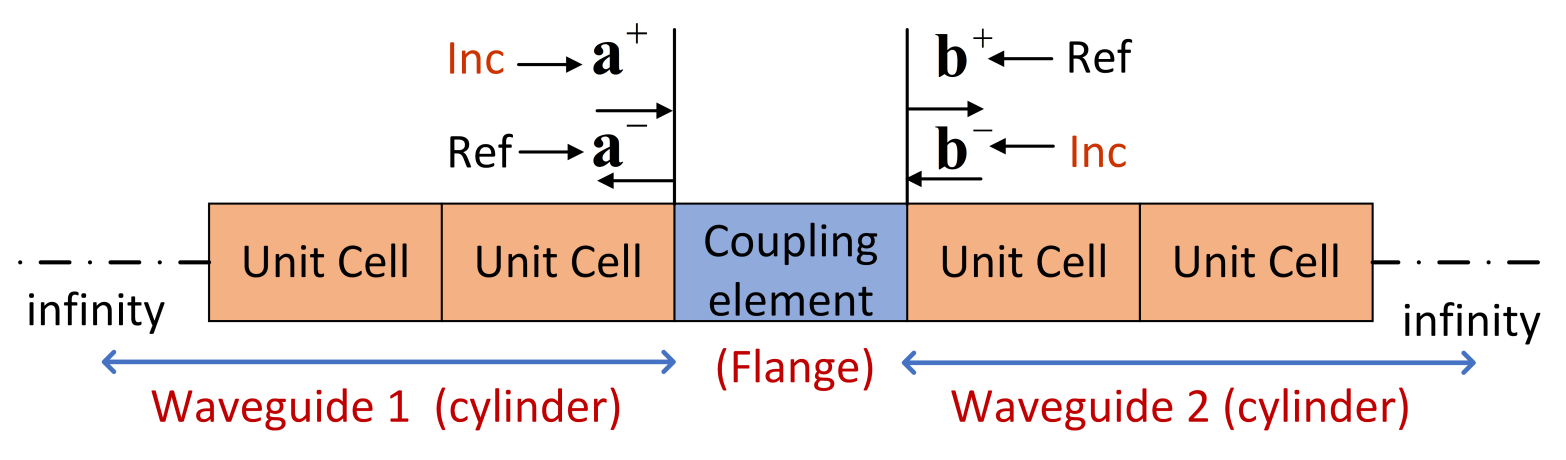
Wave-scattering of the flange connected to the cylindrical shell. Incident waves $a^{+}$ and $b^{-}$ enter couple element and reflect respective waves $a^{-}$ and $b^{+}$.

The diffusion matrix method [16,17] is a powerful numerical tool in modeling and wave-scattering calculation, especially for the local damage in this paper. It extracts the dynamic stiffness matrix of a coupling element from the FEM model and decomposes the displacements and forces of boundary DOFs into the wave domain. As shown in Figure 3, a coupling element is introduced into an infinite periodic structure of the cylinder. The physical motions q and f of a cross-section of waveguide 1 or 2 can be decomposed into the wave domain, with the following expression:

(8) $$\left[\begin{array}{c} q\\ f \end{array}\right]=\Phi \cdot Q=\left[\begin{array}{cc} {\Phi_{q}}^{+} & {\Phi_{q}}^{-}\\ {\Phi_{f}}^{+} & {\Phi_{f}}^{-} \end{array}\right]\left[\begin{array}{c} Q^{+}\\ Q^{-} \end{array}\right],$$

where Q is the vector (N×1) of wave amplitudes. The dynamical equilibrium of the coupling element can be expressed as Equation (2):

(9) $${\tilde{D}}^{\left(c\right)}\left[\begin{array}{c} q_{L}^{\left(c\right)}\\ q_{R}^{\left(c\right)} \end{array}\right]=\left[\begin{array}{c} f_{L}^{\left(c\right)}\\ f_{R}^{\left(c\right)} \end{array}\right].$$

Considering the displacement continuity and force equilibrium condition of the left and right cross-profiles of the coupling element, it can be obtained that

(10) $$\begin{array}{cc} \left[\begin{array}{c} q_{L}^{\left(c\right)}\\ q_{R}^{\left(c\right)} \end{array}\right]=\left[\begin{array}{c} q^{\left(1\right)}\\ q^{\left(2\right)} \end{array}\right] & \left[\begin{array}{c} f_{L}^{\left(c\right)}\\ f_{R}^{\left(c\right)} \end{array}\right]=\left[\begin{array}{c} f^{\left(1\right)}\\ -f^{\left(2\right)} \end{array}\right] \end{array}.$$

Suppose that $a^{+}$ and $b^{-}$ are incident waves, and the reflected waves are $a^{-}$ and $b^{+}$. By introducing Equation (10) into Equation (9) and expressing Equation (9) in the wave domain, we obtain

(11) $${\tilde{D}}^{\left(c\right)}\left[\begin{array}{cc} \Psi_{q}^{\mathrm{ref}} & \Psi_{q}^{\mathrm{inc}} \end{array}\right]\left[\begin{array}{c} a^{-}\\ b^{+}\\ a^{+}\\ b^{-} \end{array}\right]=\left[\begin{array}{cc} \Psi_{f}^{\mathrm{ref}} & \Psi_{f}^{\mathrm{inc}} \end{array}\right]\left[\begin{array}{c} a^{-}\\ b^{+}\\ a^{+}\\ b^{-} \end{array}\right],$$

where

$$\begin{array}{c} \begin{array}{cc} \Psi_{q}^{\mathrm{ref}}=\left[\begin{array}{cc} \Phi_{q}^{\left(1-\right)} & 0\\ 0 & \Phi_{q}^{\left(2+\right)} \end{array}\right] & \Psi_{q}^{\mathrm{inc}}=\left[\begin{array}{cc} \Phi_{q}^{\left(1+\right)} & 0\\ 0 & \Phi_{q}^{\left(2-\right)} \end{array}\right] \end{array}\\ \begin{array}{cc} \Psi_{f}^{\mathrm{inc}}=\left[\begin{array}{cc} \Phi_{f}^{\left(1+\right)} & 0\\ 0 & -\Phi_{f}^{\left(2-\right)} \end{array}\right] & \Psi_{f}^{\mathrm{inc}}=\left[\begin{array}{cc} \Phi_{f}^{\left(1+\right)} & 0\\ 0 & -\Phi_{f}^{\left(2-\right)} \end{array}\right] \end{array}. \end{array}$$

The relationship between the incident and reflected waves can be obtained by rewriting Equation (11) as

(12) $$\left[\begin{array}{c} a^{-}\\ b^{+} \end{array}\right]=C\left[\begin{array}{c} a^{+}\\ b^{-} \end{array}\right],$$

where

$$C={\left({\tilde{D}}^{\left(c\right)}\Psi_{q}^{\mathrm{ref}}-\Psi_{f}^{\mathrm{ref}}\right)}^{-1}\left(-{\tilde{D}}^{\left(c\right)}\Psi_{q}^{\mathrm{inc}}+\Psi_{f}^{\mathrm{inc}}\right)$$

C is also called a diffusion matrix [16,17]. When a reduced wave basis is retained, i.e., Φ is $n\times m\left(n>m\right)$, we can pre-multiply a matrix constructed from the left eigenvectors, such as

$$\Pi =\left[\begin{array}{cc} \Theta_{q}^{\left(1+\right)} & 0\\ 0 & \Theta_{q}^{\left(2+\right)}, \end{array}\right]$$

to avoid searching for pseudo-inverses of the non-square matrix in Equation (12). The diffusion matrix can be written as

(13) $$C={\left(\Pi {\tilde{D}}^{\left(c\right)}\Psi_{q}^{\mathrm{ref}}-\Pi \Psi_{f}^{\mathrm{ref}}\right)}^{-1}\left(-\Pi {\tilde{D}}^{\left(c\right)}\Psi_{q}^{\mathrm{inc}}+\Pi \Psi_{f}^{\mathrm{inc}}\right).$$

### 3.3. Verification

**Figure 4:**
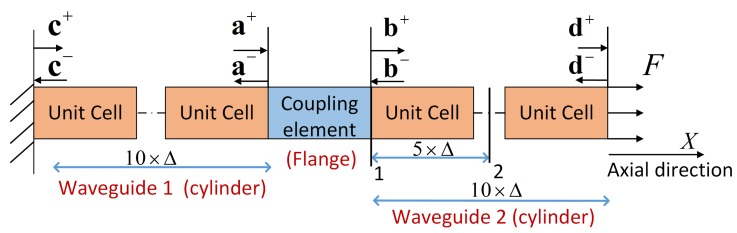
Forced response analysis model in WFEM and FEM. The left boundary is clamped, and a force of 100*N* and 132kHz is applied at the right boundary. The axial displacements of cross-profiles 1 and 2 are extracted and illustrated in Figure 6.

**Figure 5:**
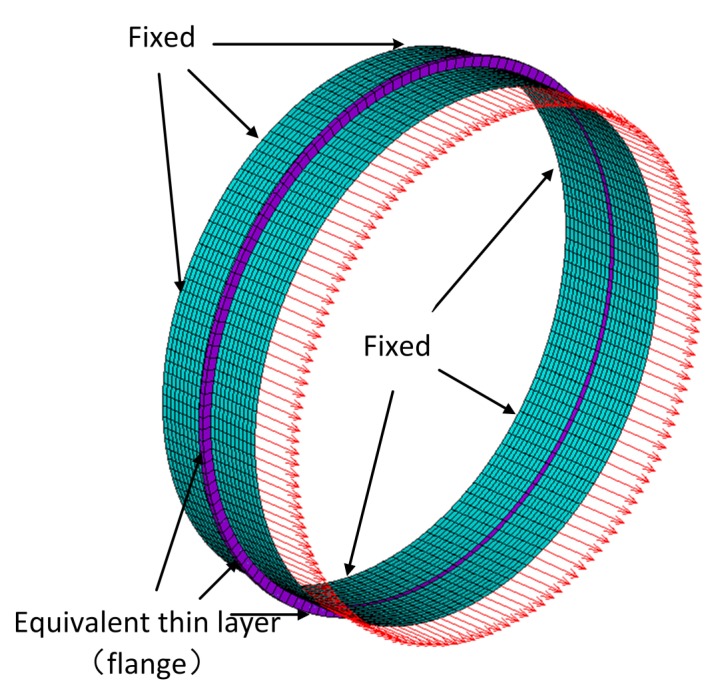
Forced response analysis model of Figure 4 shown in ANSYS.

We conduct a numerical simulation to apply the DMM method on the flange joint. As shown in Figure 4 and Figure 5, fixed and force boundaries are respectively applied to the left and right cross sections of 10 unit cells (cylinder) away from the coupling element. Force response analysis is subsequently implemented in FEM and WFEM. Assuming waves a and b propagate through 10-unit cells in the positive and negative direction, wave c and wave d are written as

(14) $$\begin{array}{cc} c={\left(\Lambda^{\left(1\right)}\right)}^{-10}a & d={\left(\Lambda^{\left(2\right)}\right)}^{10}b \end{array}.$$

The fixed boundary and force boundary are respectively

(15) $$\begin{array}{cc} \Phi_{q}^{\left(1\right)}c=0 & \Phi_{f}^{\left(2\right)}d=F \end{array}.$$

**Figure 6:**
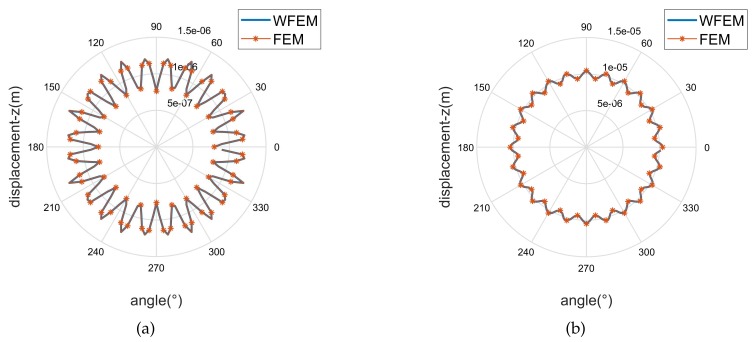
Axial displacement of cross-profiles 1 and 2 calculated in WFEM and FEM.

Considering Equations (14) and (15) and Equation (12), a, b, c, and d can be solved. As an example, the excitation frequency is 132kHz, as given in Section 5.2, and the axial force of 100*N* is applied at the right end. The geometric features and material parameters of the unit cell and coupling element are given in Section 2 and Section 4.1, respectively. Considering the influence of evanescent waves, the axial displacements of cross-profiles 1 and 2 are extracted, and Figure 6 illustrates the results in WFEM and FEM. Reasonable agreement can be observed, and the average relative error is 0.4% and 0.91%, respectively, when adjusting the retaining wave basis in $0.09<\left|\lambda \right|<11.11$. Under this condition, all the propagating waves and 380 evanescent waves are retained in the wave basis. Please note that the number of waves in the reduced wave basis depends on the frequency band and should be examined by this procedure at every applied frequency.

## 4. Wave-Scattering Characteristic in Cylinder and Joint

### 4.1. Dispersive Curves of Cylinder

**Figure 7:**
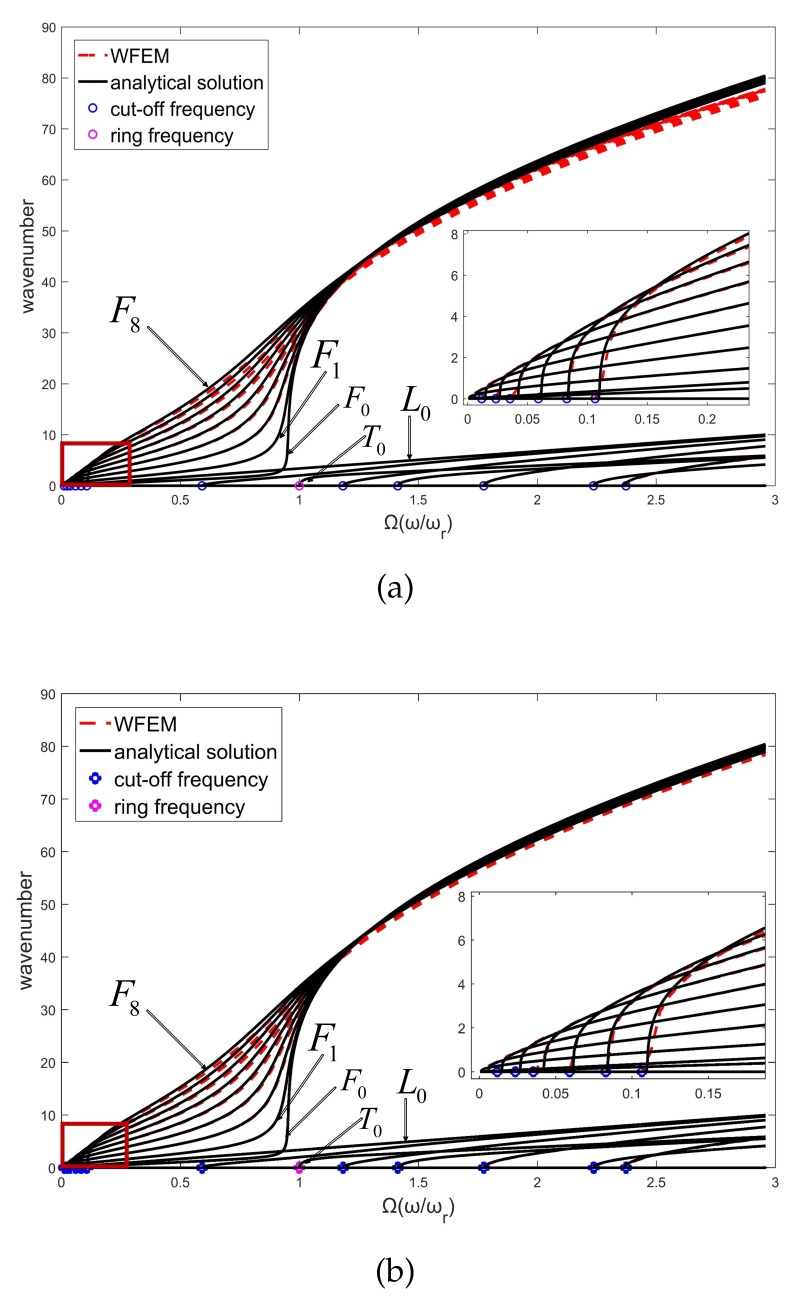
Dispersive curves of a cylinder in axial lengths of (**a**) 0.01 m, and (**b**) 0.005 m, obtained by WFEM and high-order analytical wave equation. The bottom shows longitudinal and torsion waves, and the top shows flexural waves.

**Figure 8:**
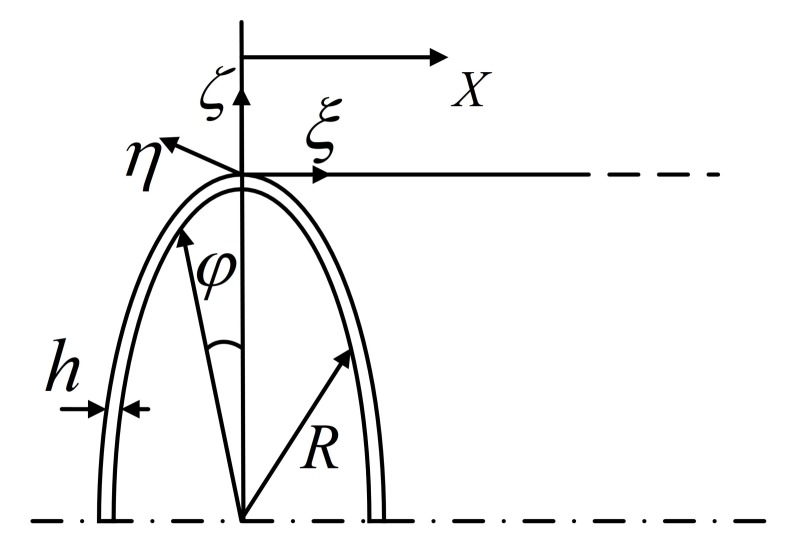
Cylindrical coordinate system: ω, ξ, and η are radial, circumferential, and axial displacements, respectively.

**Figure 9:**
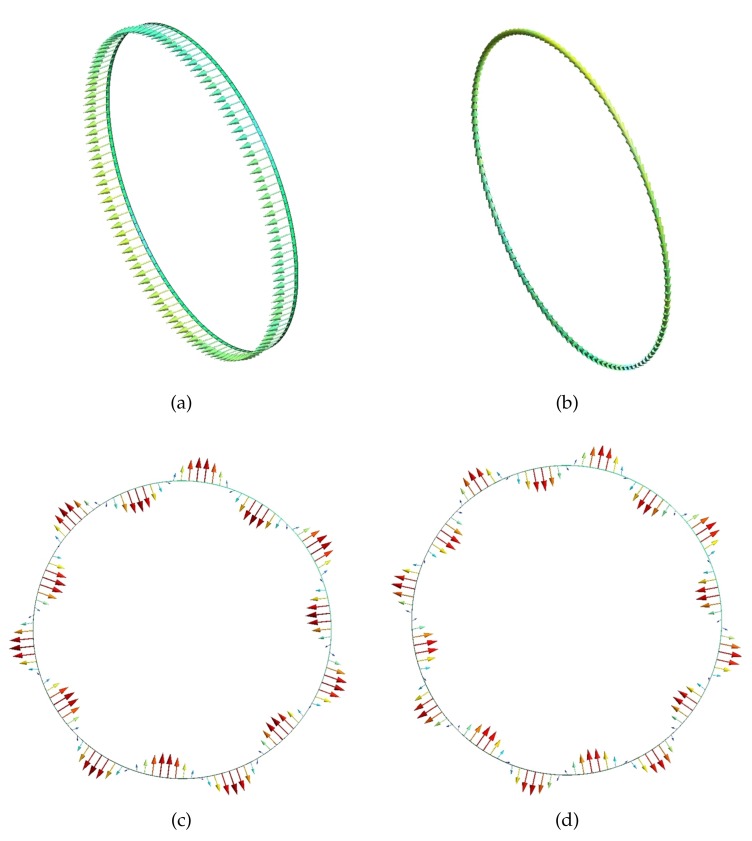
Wave-shapes calculated by WFEM at 1000 Hz shown in Gmesh: (**a**) $L_{0}$; (**b**) $T_{0}$; (**c**) $F_{7}$; (**d**) $F_{8}$.

The cylindrical waveguide in Section 2 is divided into unit cells with axial lengths of 0.01 m and 0.005 m. Material parameters of the cylinder are assumed to be E=2.1·1011NNmm, ρ=7.8·103KgKgm3m3, and ν=0.3. SHELL181 is used to model the unit cell in FEM, and the number of circumferential grids is 120. The dispersive curves calculated by WFEM are shown in Figure 7 by red dashed lines. The black solid lines are the analytical solution calculated by the high-order wave equation of a thin-walled cylindrical shell [32,33]. The abscissa is the normalized frequency [34],

Ω=ωωωrωr

where $\omega_{r}$ is the ring frequency (The ring frequency is the cutoff frequency when the circumferential wavenumber *n* is 0, and it has a ring wave-shape.) of the cylinder. With the frequency increasing, various evanescent waves transform to propagating waves through their cutoff frequencies. (The cutoff frequency is the demarcation point of a wave between propagating mode and evanescent mode.) For clarity, several representative waves are shown in Figure 7. These selected waves can be distinguished by projecting their wave-shapes to the normalized wave-shapes of the analytical solution, which can be expressed as

(16) $$\Phi_{n}=A_{n}\Phi_{L_{n}}+B_{n}\Phi_{T_{n}}+C_{n}\Phi_{F_{n}},$$

where

$$\begin{array}{ccc} \Phi_{L_{n}}=\left[\begin{array}{c} \zeta \\ \xi \\ \eta \end{array}\right]=\left[\begin{array}{c} 0\\ \cos\left(n\varphi \right)\\ 0 \end{array}\right] & \Phi_{T_{n}}=\left[\begin{array}{c} 0\\ 0\\ \cos\left(n\varphi \right) \end{array}\right] & \Phi_{F_{n}}=\left[\begin{array}{c} \sin\left(n\varphi \right)\\ 0\\ 0 \end{array}\right], \end{array}$$

where *L*, *T*, and *F* indicate longitudinal, torsional, and flexural waves; $A_{n}$, $B_{n}$, and $C_{n}$ are wave amplitudes of these three waves and are normalized by $A_{n}^{2}+B_{n}^{2}+C_{n}^{2}=1$; ζ, ξ, and η are respectively radial, circumferential, and axial displacements, as shown in the cylindrical coordinate system in Figure 8; and *n* is the wavenumber along the circumferential direction. For brevity, a longitudinal wave with circumferential wavenumber 0 is abbreviated as $L_{0}$, and similarly for other waves. The biggest numbers in the projecting results, i.e., $A_{n}$, $B_{n}$, and $C_{n}$, represent the modes of the numerical waves. By introducing wave-shape vectors in Gmesh, Figure 9a–d illustrate the wave-shapes of $L_{0}$, $T_{0}$, $F_{7}$, and $F_{8}$ at 1000 Hz.

**Figure 10:**
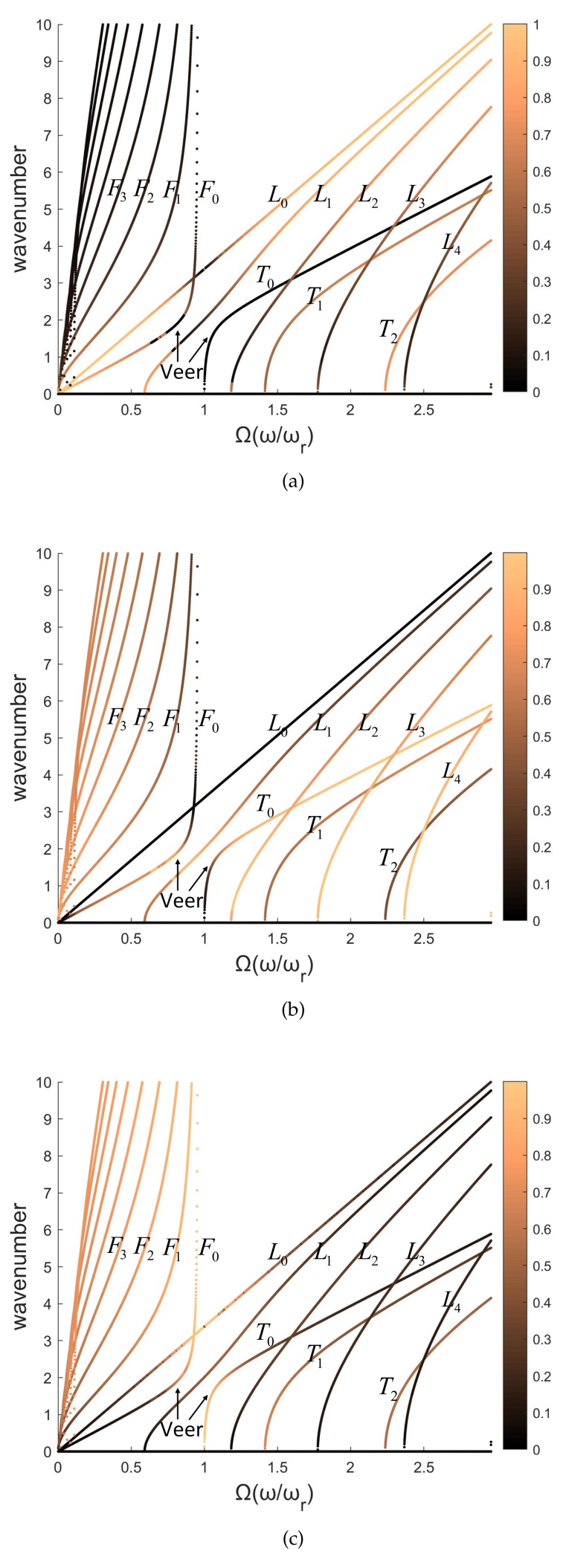
Amplitudes in Equation (16): (**a**) $A_{n}$, (**b**) $B_{n}$, and (**c**) $C_{n}$ of an *n*-order wave mode. These amplitudes represent the weights of wave-shapes of $L_{n}$, $T_{n}$, $F_{n}$, and are normalized by $A_{n}^{2}+B_{n}^{2}+C_{n}^{2}=1$.

Please note that the waves are coupling in three directions, especially near the cutoff frequencies. Figure 10a–d illustrate the amplitudes of three vibration directions of analytical wave-shapes. As the frequency increases, the coupling wave modes rapidly evolve into a single wave mode, and then the veer of dispersive curves appears, which is highly significant on $F_{0}$ and $T_{0}$ waves. In this paper, the selected wave modes work far from their cutoff frequencies to avoid the coupling modes.

The dispersive curves at the bottom of Figure 7 are longitudinal (n≤5) and torsion waves (n≤3), and at the top are flexural waves (n≤8). Good agreement can be observed between numerical and analytical results when *n* is small, such as for $L_{0}$, $T_{0}$, and $F_{0}$. However, as *n* of flexural waves increases from 0 to 8, the numerical error gradually increases in the low-frequency band (0–1) due to discretization error in the circumferential direction of the cross-section. Comparing Figure 7a,b, the axial grids of 0.005 m perform better than those of 0.01 m. This indicates that the discretization error in the axial direction induces the numerical error at high frequencies (2–3). A minimum 10 nodes per wavelength are recommended [35,36] with 120 circumferential grids, which can depict 0–12 wave modes. Conversions between these wave modes are considered in the next section.

### 4.2. Wave-Mode Conversion Induced by Local Damage of the Joint

**Figure 11:**
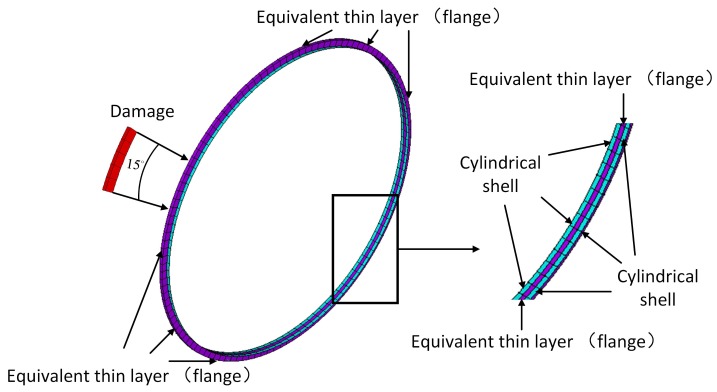
FEM model of a coupling element. The damage is induced by reducing 10/50% Young’s modulus of the thin-layer element at 0–15 degree. The blue part is the cylindrical shell; the purple part is the equivalent thin layer.

**Figure 12:**
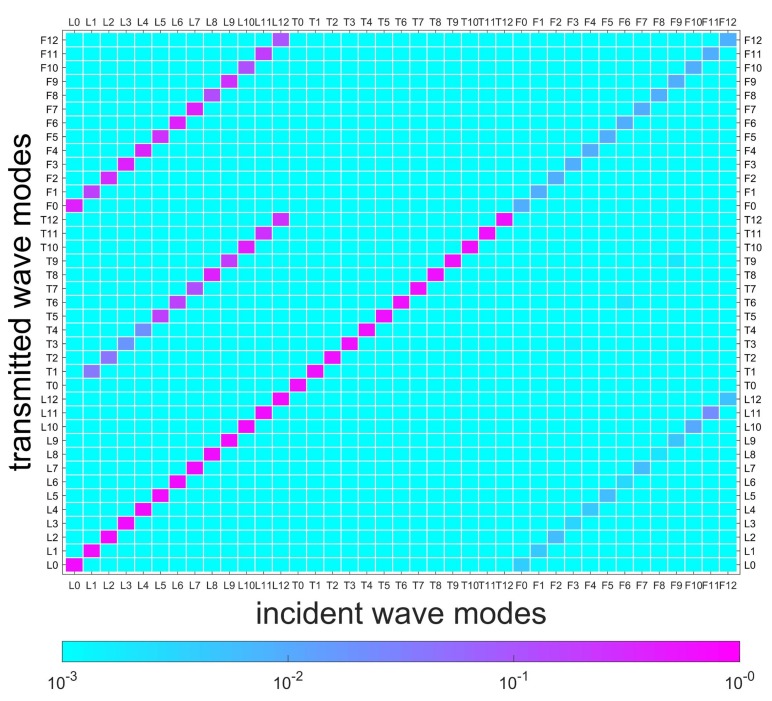
Transmitted waves of the coupling element without damage. The cool color-bar indicates the wave amplitude in the logarithmic coordinate system. The wave amplitudes of conversions higher than $10^{-3}$ are shown and marked.

Figure 11 illustrates the FEM model of a coupling element, including the equivalent thin layer of the flange and its connected cylindrical waveguide. The red elements are used to simulate local stiffness reduction of the damaged position. Material parameters of the layer are assumed to be E=1.8·1011NNmm,ρ=7.8·103KgKgm3m3,v=0.3. SOLID185 is used to model the layer, and the number of circumferential grids is 120. Assuming no wave $b^{-}$ exists in Figure 3, wave modes $L_{0-12}$, $T_{0-12}$, and $F_{0-12}$ in wave $a^{+}$ are selected from the reduced wave basis by projecting all the wave-shapes to the analytical wave-shapes of 0–12 orders in Section 4.1, and then successively actuated to the coupling element at their working frequencies, as given in Section 5.2. Please note that the reduced wave basis contains more than the wave modes of 0–12 orders; the retained rule is demonstrated in Section 3.3. A result matrix of 39×39, in which the rows and columns are incident wave $a^{+}$ and transmitted wave $b^{+}$, respectively, is obtained, and is shown in Figure 12. The cool color-bar indicates the wave amplitudes in the logarithmic coordinate system.

**Figure 13:**
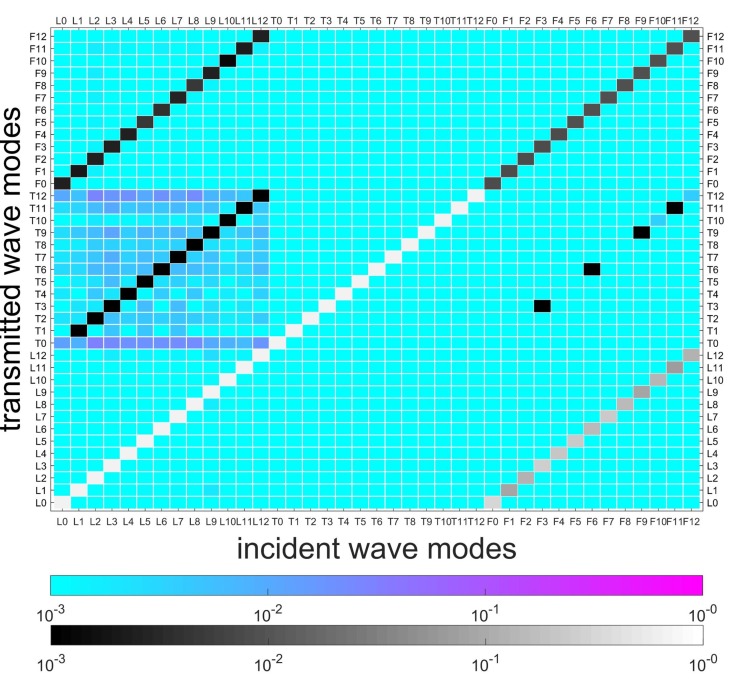
Transmitted waves of coupling element with damage (10% Young’s modulus loss in $15^{\circ }$). The marked conversions in Figure 12 are caused by both the flange and damage in this case. Hence, they are painted by a gray color-bar to distinguish them from wave-mode conversion induced by damage.

**Figure 14:**
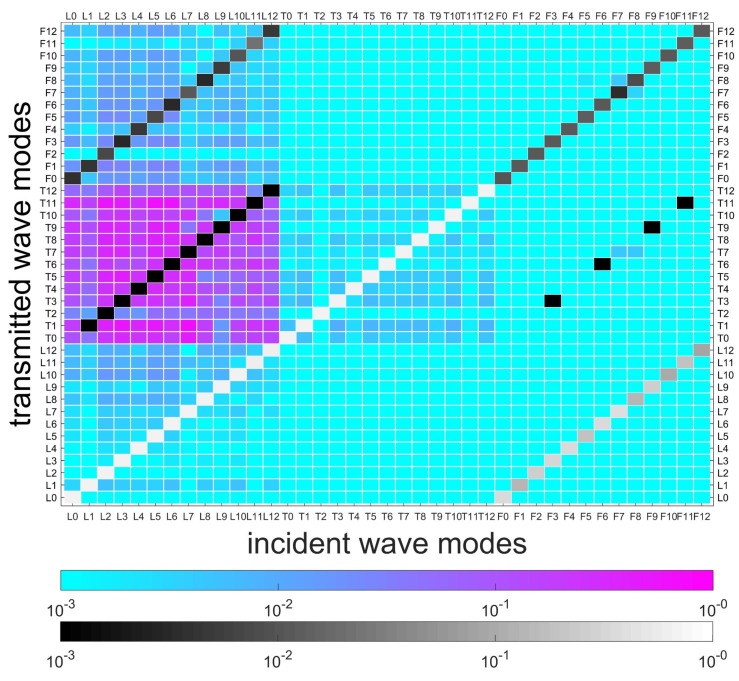
Transmitted waves of coupling element with damage (50% Young’s modulus loss in $15^{\circ }$). The marked conversions in Figure 12 are caused by both the flange and damage in this case. Hence, they are painted by a gray color-bar to distinguish them from wave-mode conversion induced by damage.

Several waves can be observed in $b^{+}$, and most wave amplitudes are less than $10^{-3}$. For visibility, Figure 12, Figure 13 and Figure 14 illustrate the details of wave amplitudes that are higher than $10^{-3}$. Due to the radial asymmetry of the flange with respect to the shell, longitudinal waves yield torsional and flexural waves of the same circumferential wavenumber *n*. This indicates the feature of SHM for joints that the intact joint can inherently cause original wave-mode conversion. Moreover, the diagonal elements of longitudinal and torsional waves in Figure 12 are close to 1, which suggests that these waves have a strong transmission ability. However, flexural waves are almost reflected, since their diagonal elements are much smaller. This is because flexural waves possess the feature of out-of-plane wave motion and are reflected by the relatively higher joint structure.

Two types of damage/asymmetry are successively induced by 10% and 50% elastic modulus losses of elements from $0^{\circ }$ to $15^{\circ }$ in the layer, as shown in Figure 11. In Figure 12, the conversions caused by the flange are marked, and in Figure 13 and Figure 14, these marked conversions are induced by both the flange and the damage. Hence, Figure 13 and Figure 14 paint these conversions by a gray color-bar to distinguish them from wave-mode conversions induced by the damage. When the change of connection status occurs, slight/complex wave-mode conversions of the two types of damage appear compared to the undamaged joint. This indicates that slight damage can induce wave-mode conversion, and this feature can be used to identify the damage. Additionally, in Figure 14, longitudinal waves are found to be more sensitive to the damage and yield almost all wave modes, especially the torsional waves, while torsional and flexural waves yield other wave modes to a small extent.

This section calculates the wave-mode conversion of the flange joint with damages, and we will next discuss the transducers to identify these wave modes.

## 5. Piezoelectric Transducers for Waves in Cylinders

### 5.1. Determining the Distribution of Electrodes

**Figure 15:**
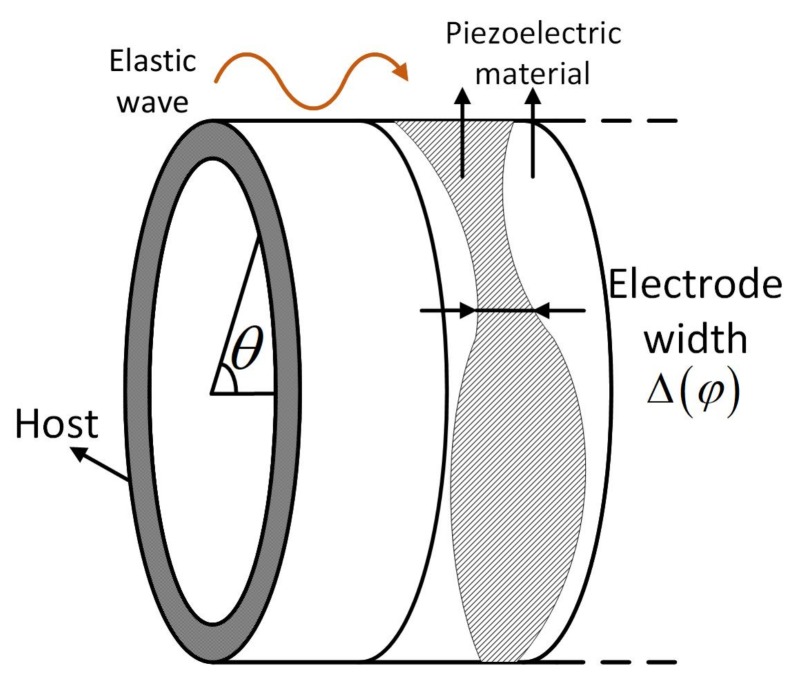
Transducer implemented on host structure. Piezoelectric material is extended along the circumferential direction. Hatched area is polarized.

The desired piezoelectric transducers are those that can only couple with one wave. As shown in Figure 15, the piezoelectric material is placed along the circumferential direction of the cylinder, and the electrode width $\Delta \left(\varphi \right)$ acts like a filter function. Consequently, we can use them to extract the weight of such a wave mode from the overall displacement field of a cylinder, which is the weighted superposition of all the waves,

(17) $$u=\left[\begin{array}{c} \zeta \\ \xi \\ \eta \end{array}\right]=a_{L,n}\Phi_{L,n}+a_{T,n}\Phi_{T,n}+a_{F,n}\Phi_{F,n},$$

where u is the displacement field. The voltage response of the piezoelectric transducer is

(18) $$V\left(u\right)=a_{L,n}v\left(\Phi_{L,n}\right)+a_{T,n}v\left(\Phi_{T,n}\right)+a_{F,n}v\left(\Phi_{F,n}\right),$$

where *v* is the voltage induced by $L_{n}$, $T_{n}$, and $F_{n}$. Through appropriate distribution and interconnection of the piezoelectric materials, the function v() can yield significant value only for one wave yet return minor values for the other waves.

**Figure 16:**
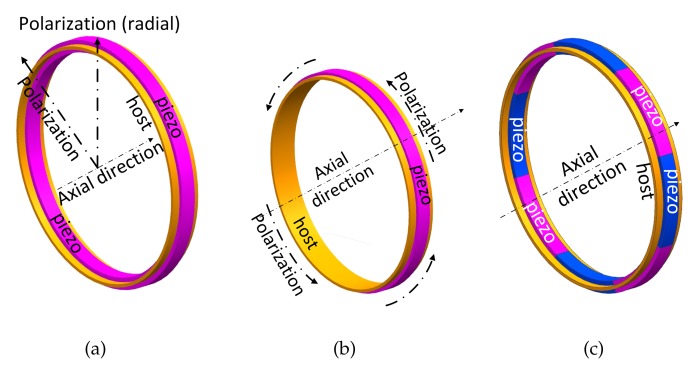
Transducer designs for (a) $L_{0}$, (b) $T_{0}$, and (c) $F_{4}$. (**a**) double layer, radial polarization. (**b**) single layer, circumferential polarization. (**c**) double layer, radial polarization; different colors indicate reverse polarization.

**Figure 17:**
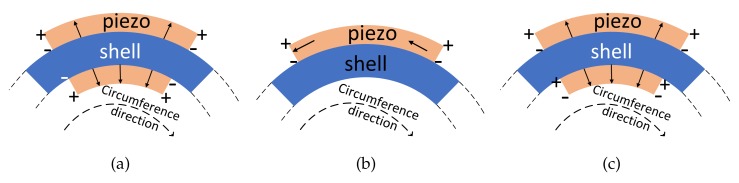
Circuit connection of transducer with radius increasing. (**a**) +--+; (**b**) -+; (**c**) -+-+.

Let us first discuss the design of transducers for the first-order longitudinal wave. In this case, the polarization direction of piezoelectric materials is parallel to the radial direction, as shown in Figure 16a and Figure 17a. The voltage induced by a longitudinal wave can be written as

(19) $$V_{r}=\frac{Q_{r}}{C}=\frac{1}{C}{\;\int \int \;}_{s}D_{r}ds,$$

where $Q_{r}$ is the quantity of electricity, *C* is the intrinsic capacitance, $D_{r}$ is the electric displacement in the radial direction, and *s* is the area of the piezoelectric material at one radius. The final expression of $V_{r}$ is

(20) $$V_{r}=\sum_{n=0}^{\infty }\kappa_{n}\int_{0}^{2\pi }\Delta \left(\varphi \right)d_{31}\varepsilon_{n}\left(\varphi \right)d\varphi ,$$

where $\kappa_{n}$ is a constant associated with each wave mode, $\Delta \left(\varphi \right)$ is the electro width function in the axial direction, and $\varepsilon_{n}$ is the strain field induced by the propagation of a wave with circumferential wave number *n*.

For a given longitudinal wave, i.e., fixed *n*, if the electro width function also follows the form of the cosine function, i.e., $\Delta \left(\varphi \right)=\Delta_{0}\cos\left(m\varphi \right)$, then it can be proved that $V_{r}$ is nonzero only when n=m by the orthogonal relations of the cosine functions. This implies that the electro width function acts as a filter function that endows the transducer with a selective coupling capacity for the desired longitudinal wave (with circumferential wave number n=m). Figure 16a shows the transducer for the first-order longitudinal wave, where only a constant length is required. Please note that the torsional wave modes induce zero voltage for the longitudinal transducers, due to the radial polarization direction. The voltage induced by the flexural waves is canceled by the collocated piezoelectric materials. As shown in Figure 17a, the circuit connection of piezo is +--+ with increasing radius. For these reasons, we can prove that the transducer with the configuration shown in Figure 16a is only coupled with the first-order longitudinal wave.

**Figure 18:**
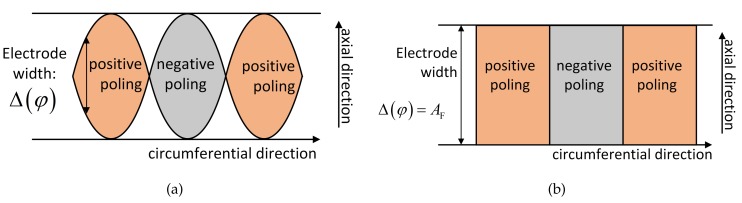
Distributed function Δ(φ): (**a**) sinusoidal function; (**b**) piecewise function.

This procedure is also applied when designing transducers for $L_{n}$ and $F_{n}$. The transducer for the first-order torsional wave is shown in Figure 16b and Figure 17b, where the poling is along the circumferential direction. As for flexural waves, the poling is also along the radial direction, and the electrode connection of the inner race is contrary to the longitudinal case. This is to filter out the contribution of the longitudinal waves in the transducer output. When $F_{4}$ is considered, its associated length function is as shown in Figure 18a. Nevertheless, the sinusoidal distribution is hard to implement in both simulation and experiment. Therefore, we use a piecewise distribution as an approximation:

(21) $$\Delta \left(\varphi \right)=\left\{\begin{array}{c} \begin{array}{cc} A_{F} & \frac{2i\pi }{n}\leq \varphi <\frac{\left(2i+1\right)\pi }{n},i=0,1,2,...,n-1 \end{array}\\ \begin{array}{cc} -A_{F} & \frac{\left(2i-1\right)\pi }{n}\leq \varphi <\frac{2i\pi }{n},i=1,2,3,...,n \end{array} \end{array}\right..$$

As shown in Figure 18b, the positive and negative signs represent radial outward and inward directions, respectively. This scheme is more feasible, and it ensures orthogonality with the guided wave mode.

### 5.2. Determining the Axial Length and Working Frequency

In wave-based SHM, the wavelength should be of the same magnitude as the targeting singularities to ensure sufficient wave-scattering. To actuate one wave, the external force should be applied in a suitable area, which in this paper corresponds to the length of the transducer. In this work, the wavelength of the actuated wave is twice the length of the joint, and the length of the piezoelectric transducer equals a half-wavelength. Then the working frequency of transducers is

(22) $$2Af=c_{g},$$

where *A* is the axial length of the joint structure, which in Figure 1b is 0.02 m; $c_{g}$ is the group velocity of a guided wave. The working frequencies in the paper are listed in Table 1 and Table 2.

### 5.3. Verification

**Figure 19:**
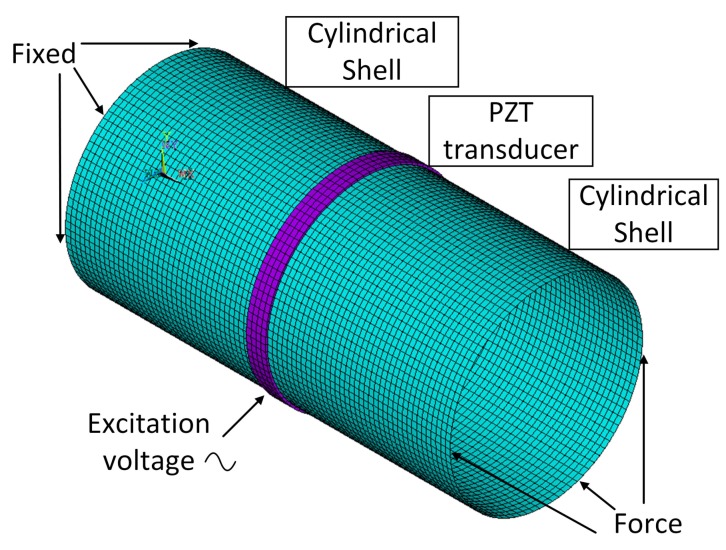
First and second verification model. SOLID5 and SHELL181 are employed for piezo and cylinder, respectively. The transducer is used as an actuator when voltage is applied, and a sensor when force is applied.

The transducers for $L_{0}$, $T_{0}$, $F_{6}$, and $F_{4}$ (abbreviated example: $L_{0}$ transducer) are modeled and analyzed by ANSYS, where the piezoelectric materials and the host cylinder are modeled by SOLID5 elements and SHELL181 elements, respectively, as shown in Figure 19. The material of the piezoelectric element is piezoelectric ceramic PZT5H, with material parameters as given in Appendix A.

The voltage DOFs of the piezoelectric transducer array are coupled to be identical by different connection patterns in Figure 17. A three-cycle sinusoidal voltage signal with a sinusoidal window, written as

(23) V=100sin(3·2πft)sin(2πft),

is applied to the positive pole and employed as a stimulant signal. The voltage of the negative pole is set to zero to simulate ground. Please note that we conduct transient analysis in the following calculations. The transient analysis can be calculated by both the WEFM method with Inverse Fourier Transform and the FEM method. However, the following FEM models have at least 5/11 segments of waveguides. This brings difficulties to re-derive the equations of the WFEM method for each FEM model. Conversely, the FEM method only needs a few steps, and can make this work easier understood and repeated. Therefore, we employ the FEM method to calculate transient responses and capture the transient signal before the waves are reflected from the boundary. This is to prevent the interference of waves from the border and simulate a realistic design in experiments.

Due to assumptions made regarding the displacement field in the thickness direction, an ‘f∗h’ problem arises when *f* becomes sufficiently large. Since guided wave propagation in the thickness direction cannot be modeled accurately, stiffening effects may be encountered, resulting in locking and overestimation of the wave velocity [37]. Therefore, wave velocity examination is employed after every wave propagation simulation to prevent the effect of these restrictions. This examination is conducted by setting the distance between the actuating point and sensing point to five wavelengths.

**Figure 20:**
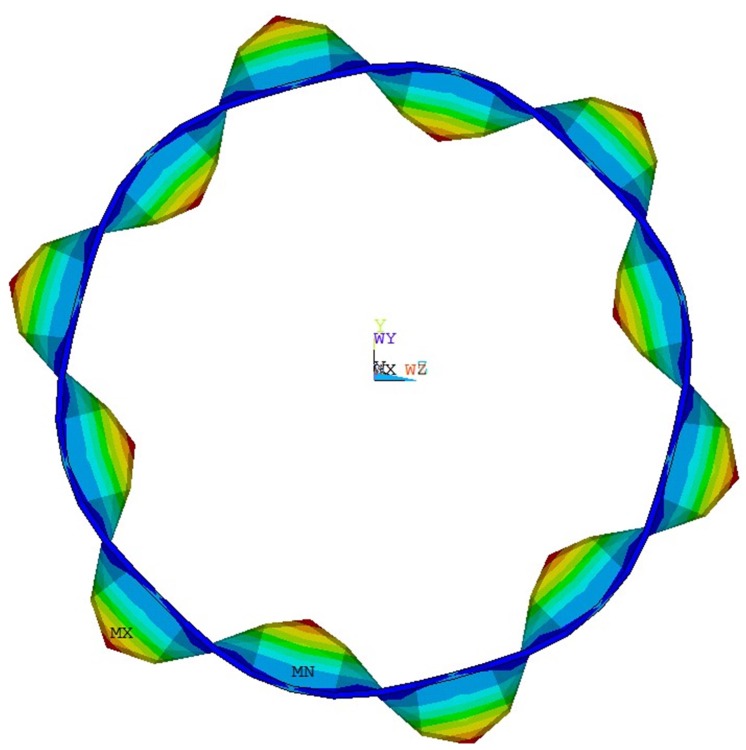
Displacement field of cylinder excited by $F_{6}$ actuator.

First, we verify the actuation capacity of the transducers by imposing a voltage on the transducer and observing the resulting displacement field. Results show that the transducers merely actuate the targeting guided wave mode. An example is shown in Figure 20, where the displacement field is actuated by a transducer designed for $F_{6}$.

**Figure 21:**
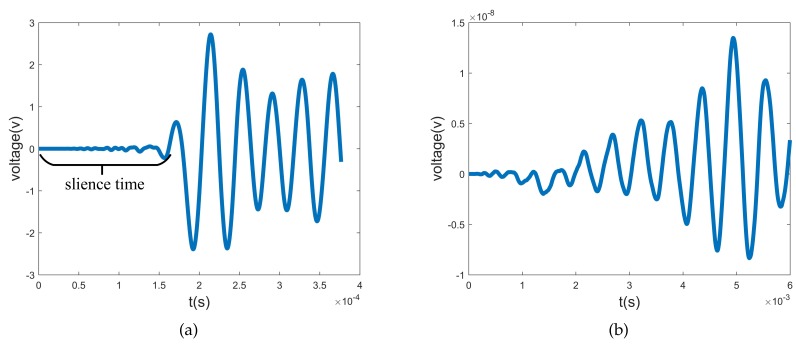
Voltage responses of $L_{0}$ sensor excited by (**a**) $L_{0}$ and (**b**) $F_{6}$ waves. The silence time area is nearly five cycles.

Second, we verify the sensing capacity of the transducers by applying the external force to the host cylinder and observing the transducer output. To test the $L_{0}$ and $T_{0}$ transducers, the forces are applied at the left end by evenly distributed sinusoidal axial force and circumferential force along the circumference. To test the $F_{6}$ and $F_{4}$ transducers, the forces applied to the left end are

(24) $$F=\sin\left(n\theta \right)\sin\left(2\pi f\cdot t\right),$$

where θ is the angle of the node in the cylindrical coordinate system. The axial length of the transducers is 0.1 m, and the working frequencies for $L_{0}$, $T_{0}$, $F_{6}$, and $F_{4}$ are listed in Table 2. The result of the $L_{0}$ transducer is shown in Figure 21. The silence time is approximately equal to five periods, and it accords with the wave velocity examination. The voltage induced by $L_{0}$ is $10^{1}V$, while those by $T_{0}$, $F_{6}$, and $F_{4}$ are $10^{-11}V$, $10^{-8}V$, and $10^{-8}V$, respectively. When the $T_{0}$, $F_{6}$, and $F_{4}$ transducers are successively implemented, differences of several orders of magnitude in voltages induced by these four wave modes can also be observed. This indicates that the piezoelectric transducer can only sense the targeting wave mode. When transducers are applied as actuators/sensors, they can be abbreviated as, for example, an $L_{0}$ actuator/sensor.

**Figure 22:**
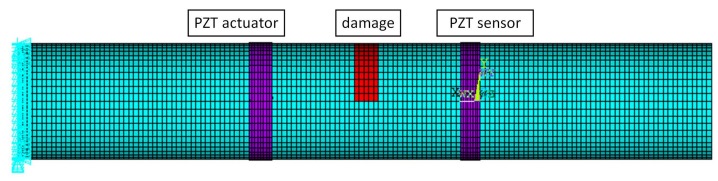
Third verification model. The left and right ends extend five wavelengths to prevent interference from reflected waves. The $L_{0}$, $T_{0}$, $F_{6}$, and $F_{4}$ transducers are successively used as actuators and sensors.

The third verification is conducted by replacing forces with piezoelectric actuators to test the actuator-damage-sensor interaction, as shown in Figure 22. The left and right ends extend five wavelengths to prevent interference from reflected waves. The axial length of the transducers is changed to 0.2 m, and Table 2 lists the working frequencies for the $L_{0}$, $T_{0}$, $F_{6}$, and $F_{4}$ sensors. Table 3 lists the voltage magnitudes of all 16 cases for the undamaged situation. The diagonal numbers are several magnitudes higher than the non-diagonal elements. This coincides with the above analysis.

**Figure 23:**
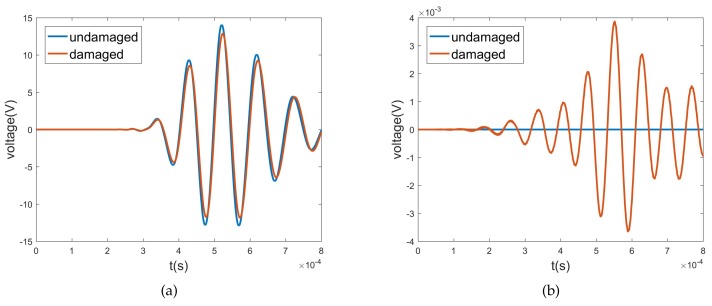
Voltage responses of (**a**) $L_{0}$, and (**b**) $F_{6}$ sensors excited by $L_{0}$ actuator.

Damage is later induced by reducing the elastic modulus of the selected elements by 50%. When $L_{0}$ is actuated by the actuator, voltage responses of $L_{0}$ and $F_{6}$ sensors are as presented in Figure 23. Compared to the undamaged cases, the voltage response of the $L_{0}$ sensor is reduced to a small extent, while that of the $F_{6}$ sensor increases from $10^{-10}$ to $10^{-3}$, i.e., a part of the lost energy in $L_{0}$ is transferred to $F_{6}$ due to interaction with damage. Results of all 16 cases are listed in Table 4. Wave-mode conversions between $L_{0}$-$F_{6}$ and $T_{0}$-$F_{4}$ are clearly observed.

These numerical simulations demonstrate that the designed wave transducers can interact with only one wave with high sensitivity. This selective electromechanical coupling feature can be used to detect small amounts of wave-mode conversion due to local damage.

## 6. Structural Health Monitoring of the Joint

**Figure 24:**
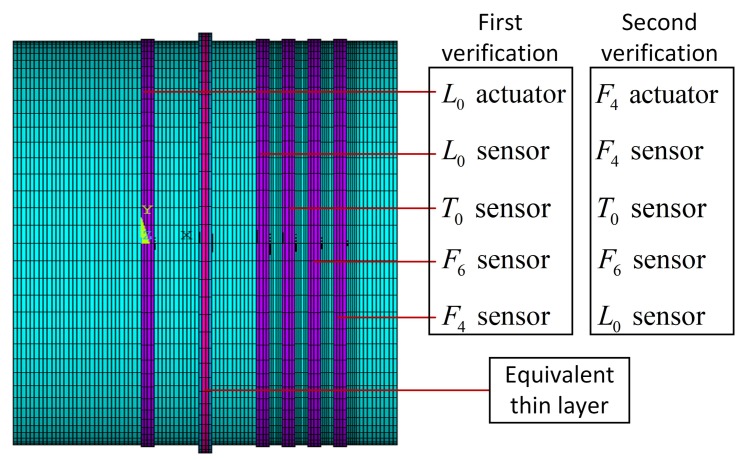
FEM model of drum-flange structure in Section 2. Actuators and sensors are implemented to detected wave-scattering features of joint structure for first and second verification.

**Figure 25:**
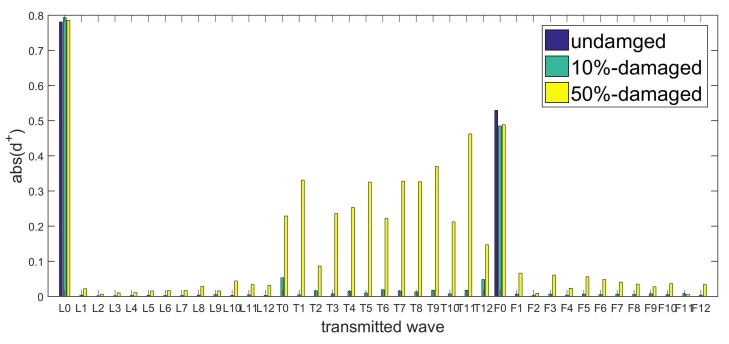
WFEM results: transmitted wave amplitudes of couple element excited by $L_{0}$.

To verify the conclusions in Section 4.2, we apply these transducers to monitor the connection status of the flange joint in Section 2, whose FEM model is shown in Figure 24. The first verification is for wave-scattering excited by longitudinal waves, because they perform better in transmission and conversion. The $L_{0}$ transducer is the simplest to implement, so it is employed as the actuator. To study transmitted waves between different families of waves and compare waves in the same family, $L_{0}$, $T_{0}$, $F_{6}$, and $F_{4}$ sensors are chosen. We redraw the $L_{0}$ columns of Figure 12, Figure 13 and Figure 14 to obtain Figure 25. Wave-mode conversion between $L_{0}$-$F_{0}$ is induced by the intact flange, and the complex conversion emerges after introducing the two types of damage.

**Figure 26:**
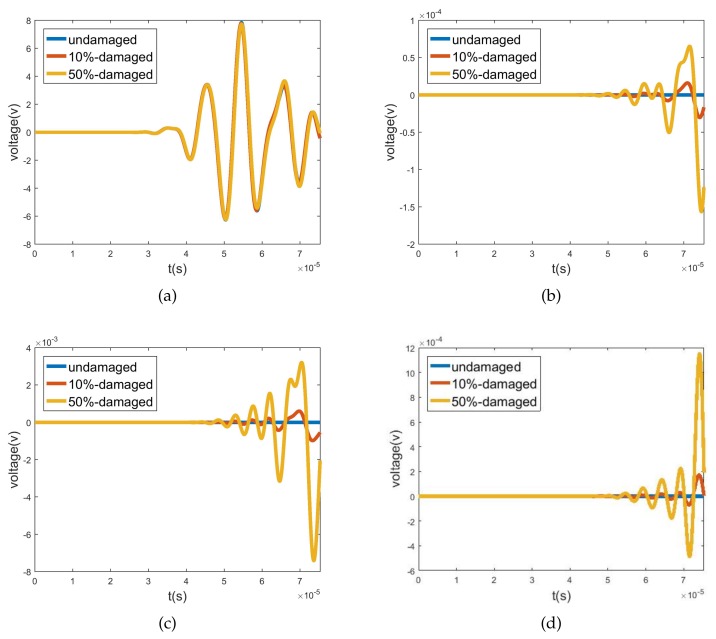
First verification: voltage responses of (**a**) $L_{0}$, (**b**) $T_{0}$, (**c**) $F_{6}$, and (**d**) $F_{4}$ sensors excited by $L_{0}$ actuator.

Transient analysis is subsequently conducted, and Figure 26a–d illustrate the voltage response of sensors excited by the $L_{0}$ actuator. When the joint is undamaged, $L_{0}$ shows a strong transmission ability and no wave conversion occurs, which is consistent with Figure 25, as the wave amplitude of $L_{0}$ is about 0.78 and the wave amplitudes of $T_{0}$, $F_{6}$, and $F_{4}$ are close to 0. After the two forms of damage are induced, the voltage of the $L_{0}$ sensor remains almost unchanged, while the voltage responses of the $T_{0}$, $F_{6}$, and $F_{4}$ sensors rise, which indicates the occurrence of wave-mode conversion between L0andT0T0F6F6F4F4F6F6F4F4. The voltages induced by 10% damage demonstrate the effectiveness of transducers for slight damage, and this is much smaller than that for 50% damage. These two points can be predicted by the WFEM results in Figure 25. Due to the differences in polarization direction and the single/double layer between torsional and flexural wave transducers, the voltages induced by the same wave are lopsided, leading to the higher voltage of the $F_{6}$ sensor in contrast to the $T_{0}$ sensor. Conversely, in flexural waves, the voltage of the $F_{6}$ sensor is higher than that of the $F_{4}$ sensor, which agrees with Figure 25.

**Figure 27:**
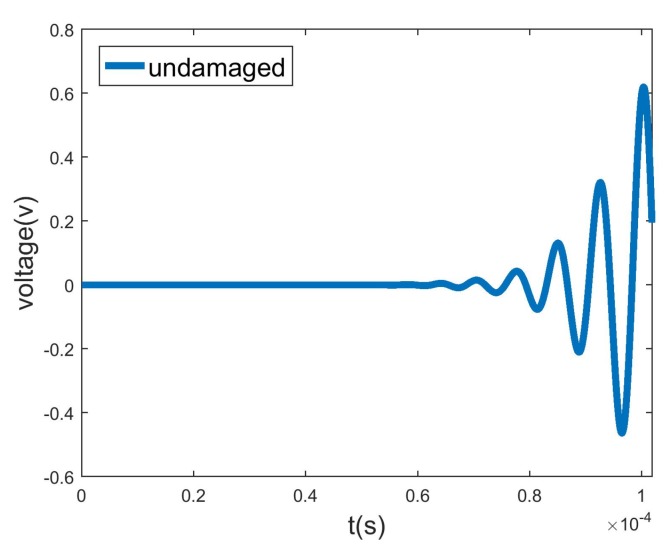
Second verification: voltage response of $F_{4}$ sensor excited by $F_{4}$ actuator.

The second verification is of the transmission ability of flexural waves, and the $F_{4}$ transducer is used as the actuator, as shown in Figure 24. To prevent the $F_{4}$ wave from being reflected by the $L_{0}$, $T_{0}$, and $F_{6}$ sensors, the $T_{0}$ and $F_{4}$ sensors swap their positions. Figure 27 shows that the voltage amplitude of the $F_{4}$ sensor excited by the $F_{4}$ actuator is 0.6*v*, which is much lower than the 8*v* of the $L_{0}$ sensor excited by the $L_{0}$ actuator. By comparing the wave amplitudes of $L_{0}$ and $F_{4}$ in the diagonal elements of Figure 12, one can conclude that both the transient result of FEM and stationary response of WEFM demonstrate that the $F_{4}$ wave is almost reflected by the flange joint.

## 7. Conclusions

In this paper, based on the wave-scattering characteristics of the joint structure calculated by WFEM, we proposed a design scheme of the distributed piezoelectric transducers in a cylindrical shell to monitor the transmitted waves. The main conclusions are as follows.

- Wave-scattering of the coupling element is first calculated by WFEM. Results of forced response analysis of DMM are consistent with FEM in that the numerical error is less than one percent. A 39×39 matrix was formed from the results of 39 incident and 39 transmitted waves, and it was found that the damaged joint (10/50% loss of elastic modulus in $15^{\circ }$) can induce slight/complicated wave-mode conversions. Longitudinal waves have a strong transmission ability and can convert almost all wave modes; flexural waves are reflected by the joint.
- Piezoelectric wave-mode transducers are then designed to detect wave modes in cylindrical waveguides. The distributed function, polarization direction, and circuit connection of transducers are designed to cancel the charge contributed from the non-targeting waves in the cylinder. This is based on orthogonality between wave-shapes. Simulation results show ten orders of magnitude difference in voltages between the targeting wave and other waves. It is demonstrated that the transducer only couples with the targeting wave.
- By implementing the actuator-joint-sensor structure, the $L_{0}$, $T_{0}$, $F_{6}$, and $F_{4}$ sensors are employed to monitor the transmitted waves of the flange induced by the $L_{0}$/$F_{4}$ actuator. $L_{0}$ is verified to have a strong transmission ability, and $F_{4}$ is almost reflected by the joint. After these two types of damage are introduced, wave-mode conversion between L0-T0T0F6F6F4F4F6F6F4F4 is detected, and the voltage comparison between the $F_{6}$ and $F_{4}$ sensors matches the result in Figure 25.
- All these numerical simulations demonstrate the promise of the designed wave-mode transducers for the SHM of joint structures. Future work includes experiments to verify the performance of transducers.

## Figures and Tables

**Table 1:** Working frequency for axial length at 0.02 m.

Wave Mode	$L_{0-12}$	$T_{0-12}$	$F_{0-12}$
working frequency	132.000 kHz	89.750 kHz	119.700 kHz

**Table 2:** Working frequency for second and third verification in Section 5.3.

	Wave Mode	$L_{0}$	$T_{0}$	$F_{6}$	$F_{4}$
Axial Length					
0.1 m		26.54 kHz	17.90 kHz	1.47 kHz	1.43 kHz
0.2 m		12.5 kHz	7.95 kHz	1.13 kHz	1.36 kHz

**Table 3:** Magnitudes of voltage responses without damage. The diagonal elements in grey color have several magnitudes higher than the non-diagonal elements.

	$L_{0}$ Sensor	$T_{0}$ Sensor	$F_{6}$ Sensor	$F_{4}$ Sensor
$L_{0}$ actuator	$10^{1}$	$10^{-9}$	$10^{-10}$	$10^{-9}$
$T_{0}$ actuator	$10^{-9}$	$10^{-2}$	$10^{-9}$	$10^{-9}$
$F_{6}$ actuator	$10^{-9}$	$10^{-8}$	$10^{0}$	$10^{-9}$
$F_{4}$ actuator	$10^{-9}$	$10^{-8}$	$10^{-9}$	$10^{0}$

**Table 4:** Magnitudes of voltage responses with damage. Grey color in Non-diagonal elements indicates wave conversions happen.

	$L_{0}$ Sensor	$T_{0}$ Sensor	$F_{6}$ Sensor	$F_{4}$ Sensor
$L_{0}$ actuator	$10^{1}$	$10^{-7}$	$10^{-3}$	$10^{-9}$
$T_{0}$ actuator	$10^{-8}$	$10^{-2}$	$10^{-9}$	$10^{-4}$
$F_{6}$ actuator	$10^{-1}$	$10^{-8}$	$10^{0}$	$10^{-8}$
$F_{4}$ actuator	$10^{-8}$	$10^{-3}$	$10^{-8}$	$10^{0}$

## Appendix A. Material properties of PZT-5H

Mass density: ρ=7500 kg/m${}^{3}$

Material stiffness matrix evaluated at constant electric field:

$$c^{E}=10^{10}\times \left[\begin{array}{cccccc} 12.6 & 7.95 & 8.41 & & & \\ 7.95 & 12.6 & 8.41 & & & \\ 8.41 & 8.41 & 11.7 & & & \\ & & & 2.3 & & \\ & & & & 2.3 & \\ & & & & & 2.35 \end{array}\right]\mathrm{Pa}$$

Permittivity matrix evaluated at constant strain:

$$\varepsilon^{S}=\varepsilon_{0}\times \left[\begin{array}{ccc} 1700 & & \\ & 1700 & \\ & & 1470 \end{array}\right]$$

where $\varepsilon_{0}=8.854\times 10^{-12}$ C/(V·m)

Piezoelectric stress coupling matrix:

$$e=\left[\begin{array}{ccc} & & -6.5\\ & & -6.5\\ & & 23.3\\ & 0 & \\ & 17 & \\ 17 & & \end{array}\right]N/\left(V\cdot m\right)$$
